# Development of novel EST-SSR markers for ploidy identification based on *de novo* transcriptome assembly for *Misgurnus anguillicaudatus*

**DOI:** 10.1371/journal.pone.0195829

**Published:** 2018-04-12

**Authors:** Bing Feng, Soojin V. Yi, Manman Zhang, Xiaoyun Zhou

**Affiliations:** 1 College of Fisheries, Key Lab of Agricultural Animal Genetics, Breeding and Reproduction, Ministry of Education, Huazhong Agricultural University, Wuhan, P. R. China; 2 School of Biology, Georgia Institute of Technology, Atlanta, GA, United States of America; National Cheng Kung University, TAIWAN

## Abstract

The co-existence of several ploidy types in natural populations makes the cyprinid loach *Misgurnus anguillicaudatus* an exciting model system to study the genetic and phenotypic consequences of ploidy variations. A first step in such effort is to identify the specific ploidy of an individual. Currently popular methods of karyotyping via cytological preparation or flow cytometry require a large amount of tissue (such as blood) samples, which can be damaging or fatal to the fishes. Here, we developed novel microsatellite markers (SSR markers) from *M*. *anguillicaudatus* and show that they can effectively discriminate ploidy using samples collected in a minimally invasive way. Specifically, we generated whole genome transcriptomes from multiple *M*. *anguillicaudatus* using the Illumina paired-end sequencing. Approximately 150 million raw reads were assembled into 76,544 non-redundant unigenes. A total of 8,194 potential SSR markers were identified. We selected 98 pairs with more than five tandem repeats for further assays. Out of 45 putative EST-SSR markers that successfully amplified and harbored polymorphism in diploids, 11 markers displayed high variability in tetraploids. We further demonstrate that a set of five EST-SSR markers selected from these are sufficient to distinguish ploidy levels, by first validating them on 69 reference specimens with known ploidy levels and then subsequently using fresh-collected 96 ploidy-unknown specimens. The results from EST-SSR markers are highly concordant with those from independent flow cytometry analysis. The novel EST-SSR markers developed here should facilitate genetic studies of polyploidy in the emerging model system *M*. *anguillicaudatus*.

## Introduction

Polyploidy occurs extensively in many groups of fishes, especially in the lower teleosts [1]. For example, several species in the genus *Misgurnus*, a member of the Cobitidae family, exhibit polyploidy and appear to tolerate ploidy levels ranging from diploid (2n = 48 or 50) to hexaploid (6n = 150) [2–4]. In the European loach *Misgurnus fossilis*, most individuals are tetraploid with 4n = 100 chromosomes [2, 5]. Recent molecular cytogenetic studies suggested that these tetraploids arose *via* an ancestral polyploidization event [6]. In some localities, a mix of triploids (3n = 75), intermediate aneuploids (87 metaphase chromosomes) and tetraploid *M*. *fossilis* were found [7].

Polyploidy is also common in the Asian loach *M*. *anguillicaudatus*. Following the first record of triploid and tetraploid individuals from a fish market [8], subsequent studies revealed its extensive polyploidy distribution. Arai [3] reported that besides the most common bisexual diploid individuals (2n = 50), a relatively high frequency of asexual diploid clones and natural triploids (3n = 75) were also observed in certain localities in Japan. Among Chinese *M*. *anguillicaudatus* populations, in addition to the most common sexual diploids, large numbers of tetraploids (4n = 100) have been recorded along the Yangtze River basin [9–11]. Experimental crosses, including induced gynogenesis [12–13], androgenesis [14], hexaploids [15], and observations of the meiotic behavior of chromosomes [16] suggest an autotetraploid origin of the natural tetraploids. Recent studies even detected rare pentaploid (5n = 125) [17] and hexaploid (6n = 150) [4] individuals in central China. As polyploid fish could gain an advantage over diploid fish through increased heterozygosity, which provide metabolic flexibility to copy with a broader array of conditions [1], the presence of multiple ploidies in *M*. *anguillicaudatus* may increase the range of environments the fish can live in. And also, owing to such natural variability at ploidy levels, *M*. *anguillicaudatus* is emerging as a promising animal model for studying genetic and phenotypic consequences of ploidy variations.

However, discriminating polyploid *M*. *anguillicaudatus* from its diploid counterparts based on morphological grounds is difficult or impossible because they are phenotypically highly similar to each other [18] and because diploids and polyploids co-exist in many natural habitats [10, 19]. Traditionally, polyploidies are determined by cytological analyses, where the numbers of chromosomes are directly counted. However, preparing for cytological counting of loach chromosome numbers is challenging and often leads to the sacrifice of the fish. Recently, flow cytometry (FCM) has been adopted for determining ploidy levels of fishes. However, FCM analysis can be only applied to fish whose size allows an invasive extraction of appropriate amount blood cells, which could damage fish [20]. In addition, these two methods are time-consuming, costly and laborious, especially when dealing with a large number of samples [21].

Given these limitations, a minimally-invasive and economical method that requires a small amount of sample could facilitate ploidy analysis of *M*. *anguillicaudatus*. In this study, we focus on microsatellite markers (SSR markers), which are popular for ploidy and pedigree analyses in fish [20–25]. Among the manifold advantages of microsatellite analysis are high reproducibility, minimal sample requirement, and the speed allowing cost-effective population genetic analyses. Microsatellite analyses have been previously applied to identifying polyploids in Amazon molly [26], silver crucian carp [21], turbot [20] and Atlantic salmon [27]. In the current study, we focus on transcriptome-derived microsatellite markers. The SSR markers developed from such data are usually referred to as EST-SSRs or genic microsatellites. EST-SSR markers are considered as superior to traditional anonymous SSR markers from genomic DNA due to their lower cost, high level of transferability among related species, and the fact that their variation may potentially inform particular phenotype traits since they are located within genes [28].

In the present study, using Illumine HiSeq 4000 platform, we sequenced liver transcriptomes of three *M*. *anguillicaudatus* and developed polymorphic microsatellite loci from the assembled non-redundant unigenes. We selected a set of five EST-SSR markers and evaluated their ability for identifying the ploidy of *M*. *anguillicaudatus*. By amplifying in 69 reference specimens and 96 ploidy-unknown specimens from a ploidy-complex population, we demonstrated the utility of the selected ploidy diagnostic EST-SSR markers in identifying the ploidy of *M*. *anguillicaudatus*.

## Materials and methods

### Ethics statement

Before each handling, the fish were anaesthetizing with tricaine methanesulfonate (MS-222) at 100 mg/L. All the experimental procedures involving fish were approved by the institution animal care and use committee of Huazhong Agricultural University. All sampling sites in this study are located in an open, abandoned field and did not involve endangered or protected species, thus, no specific permissions were required for the described field studies.

### Fish materials

Three 1-year-old diploid *M*. *anguillicaudatus* (average body weight 10.3 g), reared at the Fisheries Experimental Station of Huazhong Agricultural University, were randomly selected for RNA-Seq libraries construction. After anaesthetizing with MS-222, liver tissues were collected and flash-frozen in liquid nitrogen, then stored at -80°C prior to RNA extraction.

**Fig 1:**
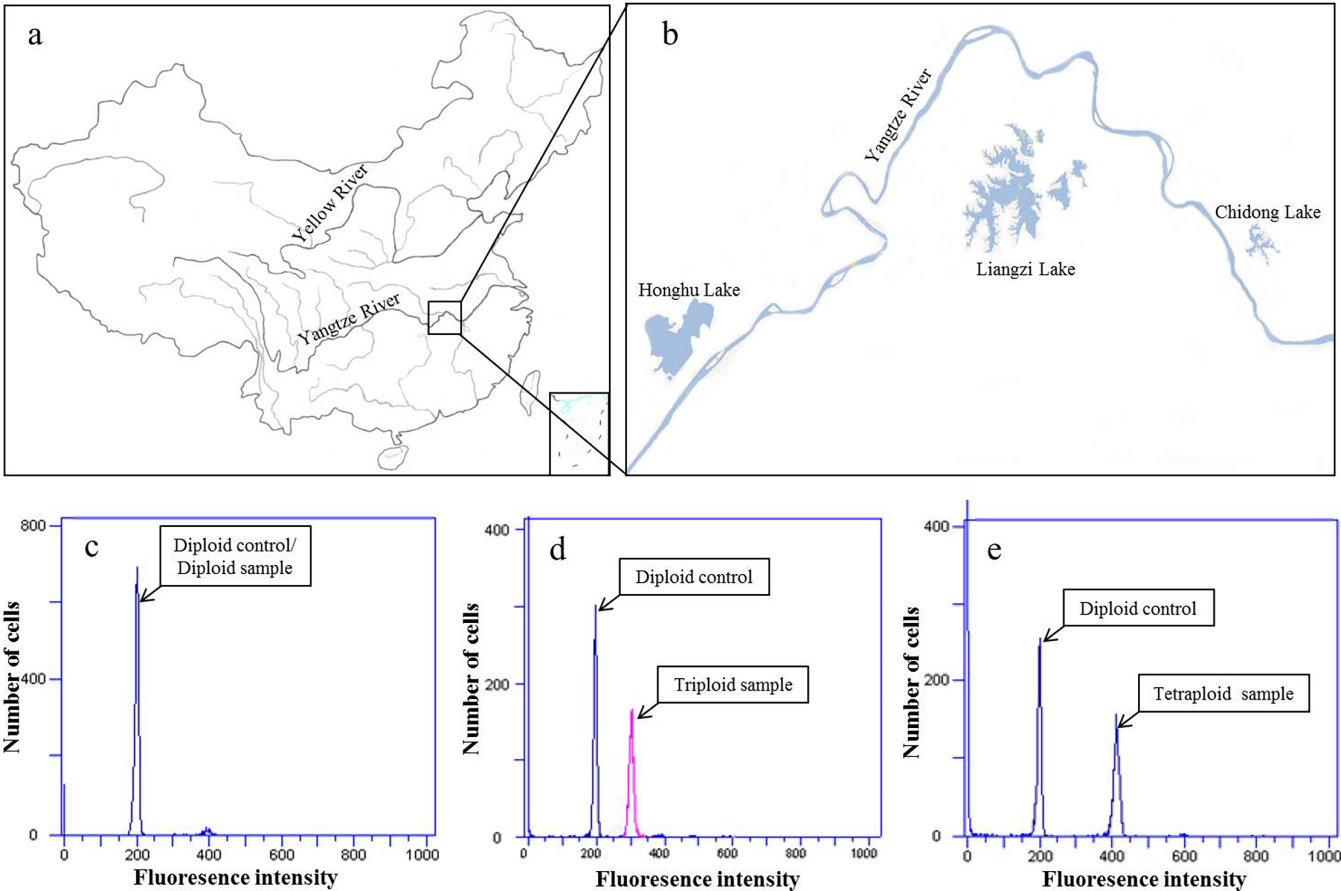
Map of China showing the river system (a) and the sampling locations (b), as well as the flow cytometry results of cellular DNA content of diploid (c), triploid (d) and tetraploid (e) *M*. *anguillicaudatus* with erythrocytes of karyotyped diploids as internal control.

Sixty-four individuals, collected from natural waters using trap nets, were initially used for validating the EST-SSR primers developed from the transcriptome sequencing. Of these individuals, 32 confirmed diploids were collected from Honghu Lake (29°49′N, 113°28′E), while 32 confirmed tetraploids were sampled from Chidong Lake (30°07′N, 115°23′E) (Fig 1). These individuals were then employed as reference specimens to evaluate the ability of EST-SSRs in ploidy identification of this species. In addition, 5 confirmed triploids obtained from Liangzi Lake (30°17′N, 114°36′E) were also examined for this purpose. The ploidy levels of these individuals were determined by analyzing the erythrocyte DNA content using a flow cytometer [29] (Fig 1).

To validate the selected ploidy diagnostic EST-SSR markers, 96 ploidy-unknown individuals were randomly collected from the Liangzi Lake (Fig 1), a locale with mixed ploidy population (primarily diploids and tetraploids) [11, 30]. For each individual, a small piece of caudal fin was cut and stored in 75% ethanol; genomic DNA was extracted using ammonium acetate method [31].

### Transcriptome sequencing and *de novo* assembly

The total RNA of each sample was extracted using TRIzol reagent (Invitrogen, USA). RNA quality and quantity were evaluated using Agilent Bioanalyzer 2100 (Palo Alto, USA) and NanoDrop 2000 spectrophotometer (Thermo Scientific, USA), respectively. RNA samples with integrity number (RIN)>8.0, 28S/18S>1.4 and OD260/280>1.8 were used for library preparation. Three cDNA libraries were generated using NEBNext^®^ Ultra RNA Library Prep Kit for Illumina^®^ (NEB, USA) following the manufacturer’s instructions. Transcriptome sequencing was performed using Illumina HiSeq^TM^ 4000 platform that generated 150 bp paired-end raw reads. All sequencing data were deposited in the NCBI Sequence Read Archive (https://trace.ncbi.nlm.nih.gov/Traces/sra) under the accession number SRP127013.

Raw reads were filtered with Trimmomatic (ver 0.30) [32] to remove adaptor contamination, unqualified reads and sequences. High quality clean reads were pooled and *de novo* assembled using Trinity [33] with default parameters, forming transcripts. Then the TIGR Gene Indices clustering tools (TGICL) (ver 2.1) [34] were used to cluster and remove redundant transcripts, and identify unigenes (i.e., non-redundant transcripts), which were used for subsequent analysis.

### EST-SSR loci identification, primer design and amplification test with diploids

MISA software (MicroSAtellite, http://pgrc.ipk-gatersleben.de/misa/) was used to scan all the unigenes for microsatellite markers. The minima of contiguous repeat units were set as mono-12, dimer-6, trimer-5, tetramer-5, pentamer-4 and hexamer-4. Primer 3 [35] was then used to design primer pairs in the flanking regions of each microsatellite locus.

Ninety-eight primer pairs with more than five tandem repeats were randomly selected, and their amplification efficacy and amplicons polymorphism were initially tested with 32 diploids. PCR amplification was performed in a 10 μL volumes containing 5 μL of 2×Taq PCR MasterMix (Qiangen, China), 0.2 μL (10 μM) of each primer and 40–50 ng template DNA. Previously studies used PCR cycles between 30–35 [20–21, 25–26]; we used 34 PCR cycles and found the resulting bands sufficiently intense and clear. PCR cycling conditions were as follows: 94°C for 10 min; 34 cycles of 94°C for 30 s, 52–56°C for 30 s, 72°C for 45 s; final extension at 72°C for 10 min. The amplified products were firstly examined with GelRed^TM^-stained (Biotium, Hayward, CA, USA) 1% agarose gels, then separated by electrophoresis on 8% non-denaturing polyacrylamide gels and visualized by silver staining.

The number of alleles (*N*_*a*_), observed heterozygosity (*H*_*o*_), expected heterozygosity (*H*_*e*_), and the frequency of null alleles (*F*_null_) at each locus were calculated using Cervus 3.0 [36]. The polymorphism information content (*PIC*) was calculated using the Excel Microsatellite Toolkit v3.1 [37], and Deviation of Hardy-Weinberg Equilibrium (*PHW*) was evaluated with the GENEPOP v 4.2 [38].

### Amplification test in tetraploids and ploidy identification EST-SSRs selection

Among the 98 primer pairs, 45 putative EST-SSR markers exhibited polymorphism in diploids (S1 Table). They were further tested in tetraploids. The *Na*, *He* and Shannon's diversity index were calculated using ATetra v1.2 [39].

SSR markers can be used for ploidy identification provided that they are variable enough to reveal as many alleles as expected for the ploidy level of the species in some loci. Thus we further evaluated the polymorphism information content (*PIC*) value [40] using the formula described in Botstein et al [41]. Following these steps, five EST-SSR primers (MATS2-33, MATS2-48, MATS2-55, MATS2-65 and MATS2-74) that amplified distinct alleles that can be easily recorded with higher *PIC* values were selected.

### Ploidy identification using EST-SSR markers

To determine the ploidy level of a candidate, it is crucial to estimate the copy number per allele. In this study, we made use of the quantitative values for microsatellite allele band intensity, provided by the Bio-Rad Quantity One^®^ software (http://www.bio-rad.com). For each locus, all alleles were quantitated according to the intensity profile of bands. Then, the alleles were analyzed in pairwise combinations to determine their copy numbers from PCR based dosage effects by calculating the ratios between quantitative values for all allele-pairs that were amplified simultaneously. For example, an individual displaying three alleles at a certain locus with the quantitative values ratios of 2:1:1, 1:2:1 or 1:1:2, we would expect it’s a tetraploids and the allele configurations of AABC, ABBC or ABCC provided that the A, B and C alleles produce similar-sized quantitative values [42]. Thus, by comparing the observed pairwise quantitative value ratios and the expected hypothetical configurations (Table 1), the allelic configuration, as well as the ploidy level of a candidate can be worked out. Furthermore, the actual number of alleles can be calculated by summing of the ratios (e.g. 2+1+1, 1+2+1 or 1+1+2), which were described as “calculated-allele numbers”, differing from the “observed-allele numbers” (the number of alleles directly recorded from electrophoretogram).

**Table 1:** Expected ratios of all possible allele configurations for diploid, triploid and tetraploid *M*. *anguillicaudatus* provided that all alleles produce similar quantitative values.

Ploidy	Diploids		Triploids				Tetraploids							
Allele patterns	One allele	Two alleles	One allele	Two alleles		Three alleles	One allele	Two alleles			Three alleles			Four alleles
Possible allele configurations	AA	AB	AAA	AAB	ABB	ABC	AAAA	AAAB	AABB	ABBB	AABC	ABBC	ABCC	ABCD
**Expected ratio**	-	1:1	-	2:1	1:2	1:1:1	-	3:1	1:1	1:3	2:1:1	1:2:1	1:1:2	1:1:1:1
**Calculated-allele numbers**	-	2	-	3	3	3	-	4	2	4	4	4	4	4

With this approach, individuals with four calculated-alleles in at least one of the five EST-SSR loci tested were considered tetraploids (and three were triploids).

### Validation of EST-SSRs for ploidy identification

The selected diagnostic EST-SSR markers were validated against 96 ploidy-unknown *M*. *anguillicaudatus* specimens collected from Liangzi Lake (Fig 1). For each individual, a small piece of caudal fin was cut for DNA extraction; while blood was collected by caudal vein puncturing and preserved in 70% alcohol at -20°C until flow cytometry analysis. The five ploidy diagnostic EST-SSR markers were used for PCR amplification. Allelic configurations were deduced according to the observed pairwise quantitative value ratios, and ploidy was estimated based on the maximum number of calculated-alleles across the five loci for each individual.

To verify ploidy levels, DNA content of each individual were measured by a BD FACSCalibur (Becton Dickinson Biosciences, San Jose, CA, USA) cytometer, using erythrocytes of karyologically identified *M*. *anguillicaudatus* with 2n = 50 as diploid control. Blood cell suspensions were prepared following to the protocol of Zhou et al [29].

## Results

### Assembly of transcriptome sequence data

A total of 149,670,620 sequence reads of 150 bps length were obtained from the three RNA-Seq libraries. After removing adaptor sequences and discarding low-quantity reads, the three sets of clean reads (53,174,150 reads for *2n_1*; 47,855,904 for *2n_2;* and 43,156,040 for *2n_3*) were merged and assembled into 223,597 transcripts using the Trinity [33]. The resulting transcripts had an average length of 938 bp and a N50 of 1,823 bp, respectively. We then clustered the transcripts using the TGICL [34], yielding 76,544 non-redundant unigenes with an average length of 877 bp and a N50 of 1,809 bp (Table 2). The length distributions of the unigenes are shown in S1 Fig.

**Table 2:** Characteristics of assembled transcripts and unigenes.

Nucleotide length (nt)	Transcripts	Unigenes
**Total number**	223,597	76,544
**Total length**	209,744,729	67,203,420
**N50 length**	1,823	1,809
**Average sequence length**	938	877
**Median length**	455	404
**200–500**	120,101	49,991
**500–1000**	41,446	11,684
**1000–2000**	32,635	7,894
**>2000**	29,384	6,944

### Detection and characterization of EST-SSRs

From the 76,544 non-redundant unigenes, a total of 8,194 potential EST-SSR loci were identified in 6,682 unigene sequences, including 1,168 unigene sequences that contained more than one EST-SSR. Of these EST-SSRs, 662 were present in compound form (with adjacent tandem simple repeats of different sequence), while 7,532 were in perfect form (without interruptions in the runs of repeat) (S2 Table). The frequency of EST-SSRs in unigenes was 10.70%, and the distribution density was one per 7.02 kb.

### Validation of EST-SSR markers in diploids

Primer pairs were designed for 5,436 loci, representing 66.34% of all EST-SSR candidate loci (S2 Table). To evaluate the amplification efficacy and amplicons polymorphism of the putative EST-SSR markers, 98 EST-SSR primer pairs with more than five tandem repeats were validated across 32 diploid individuals. We excluded mono-nucleotide repeats because which may be poly-A sequences. Among the 98 amplicons, 82 (83.67%) successfully amplified. Of the 82 successful amplicons, 80 pairs (81.63%) generated PCR products of the expected size, while other 2 (2.04%) PCR products were either shorter or longer than that expected. After the products were separated on polyacrylamide gels, 45 (45.92%) of the EST-SSR loci showed polymorphisms (S1 Table).

The number of alleles (*N*_*a*_) of those loci varied from 3 to 6, with an average of 4.0 alleles per locus and a total of 181 alleles. Estimates of the observed heterozygosity (*Ho*), expected heterozygosity (*He*), and polymorphism information content (*PIC*) ranged from 0.125 to 0.875, 0.229 to 0.799, and 0.215 to 0.755, with mean values of 0.554, 0.615 and 0.555, respectively. Detailed information for the 45 polymorphic primer pairs is shown in S1 Table.

### Variability of EST-SSRs in tetraploids

We then examined the variability of the 45 putative EST-SSRs, which were polymorphic in diploids, in tetraploid samples. We found 11 of them displaying highly polymorphism in tetraploids (Table 3). The amplification patterns were consistent with the tetraploid nature of these individuals, ranging from one to four alleles per amplified locus and individual.

**Table 3:** Characteristics of 11 EST-SSR primer pairs that amplified successfully and exhibited polymorphism in tetraploid *M*. *anguillicaudatus*.

Locus	Primer pair (5'-3')	Repeat motif	*N*_*a*_	*H*_*e*_	*PIC* value	Shannon's diversity index
**MATS1-10**	F: TGTCACAGATGTTGTAGCTCAGG R: AATAACAGAAACAACAGCAACCC	(TCA)6	6	0.686	0.625	1.375
**MATS2-22**	F: AGTGTTCTGACACCAACACAACA R: ATCAAAATTATCATGGTGATCGG	(AAT)6	7	0.809	0.777	1.760
**MATS2-33**	F: CAAACTTTAGCAGCAAAACGAGT R: TATGAAAATGTTGGTCACAGTGC	(AACA)5	7	0.803	0.765	1.719
**MATS3-42**	F: AACGAAAATCTACATTTGAGCCA R: TATCAATCCCAGAGCACTGTACC	(GA)10	6	0.775	0.717	1.596
**MATS3-48**	F: TTTGGTTTGTTTTTGTGTTGTTG R: AAGTGACAAACGGCAAATACTGT	(ACAG)6	6	0.803	0.778	1.707
**MATS3-55**	F: TATCCAACGCTTCTTCATTTCAT R: TGTATCCCCATCACAAGAAACTT	(ATG)6	8	0.810	0.773	1.781
**MATS4-65**	F: TTGTATAGAGAGCCATCTGAGCC R: ACAACACCTCACCTCTTCTGAAC	(TGA)7	7	0.815	0.785	1.760
**MATS4-74**	F: GAATGCAGAGACGTGTGAAGATA R: ACTCACTGGAGTTTCATCAGCAT	(TGA)7	7	0.819	0.792	1.800
**MATS4-75**	F: AGCAGCTTGCAGTTCAGTAGAAT R: ACGCACAAAGACACCAGAGTTTA	(ATT)7	8	0.789	0.759	1.741
**MATS5-81**	F: GAAGGGCTTAAGGAAAAGTGGTA R: GCTTCAAGCAAATAAAGCAAAGA	(CA)10	5	0.740	0.693	1.427
**MATS5-90**	F: GCAGAGTTTACCGAAAGACTGAA R: TACCATGGAGAAACACTGATGTG	(AAT)6	6	0.660	0.581	1.318
**Means**			6.64	0.774	0.731	1.635

*Na*, number of alleles; *He*, expected heterozygosity; *PIC* value, value of polymorphism information content.

Genotyping profile of these 11 loci produced allele numbers that varied from 6 to 8, with an average of 6.64. *PIC* values ranged from 0.58 to 0.79, with an average of 0.73 (Table 3). Given that an SSR with a *PIC* value > 0.5 is considered as a highly variable marker [41], the 5 EST-SSR primers (MATS2-33, MATS2-48, MATS2-55, MATS2-65 and MATS2-74) that generated distinct and easily recordable alleles with higher *PIC* values were chosen as diagnostic markers to test the ploidy levels of *M*. *anguillicaudatus* individuals.

### Ploidy identification using the diagnostic EST-SSRs

**Fig 2:**
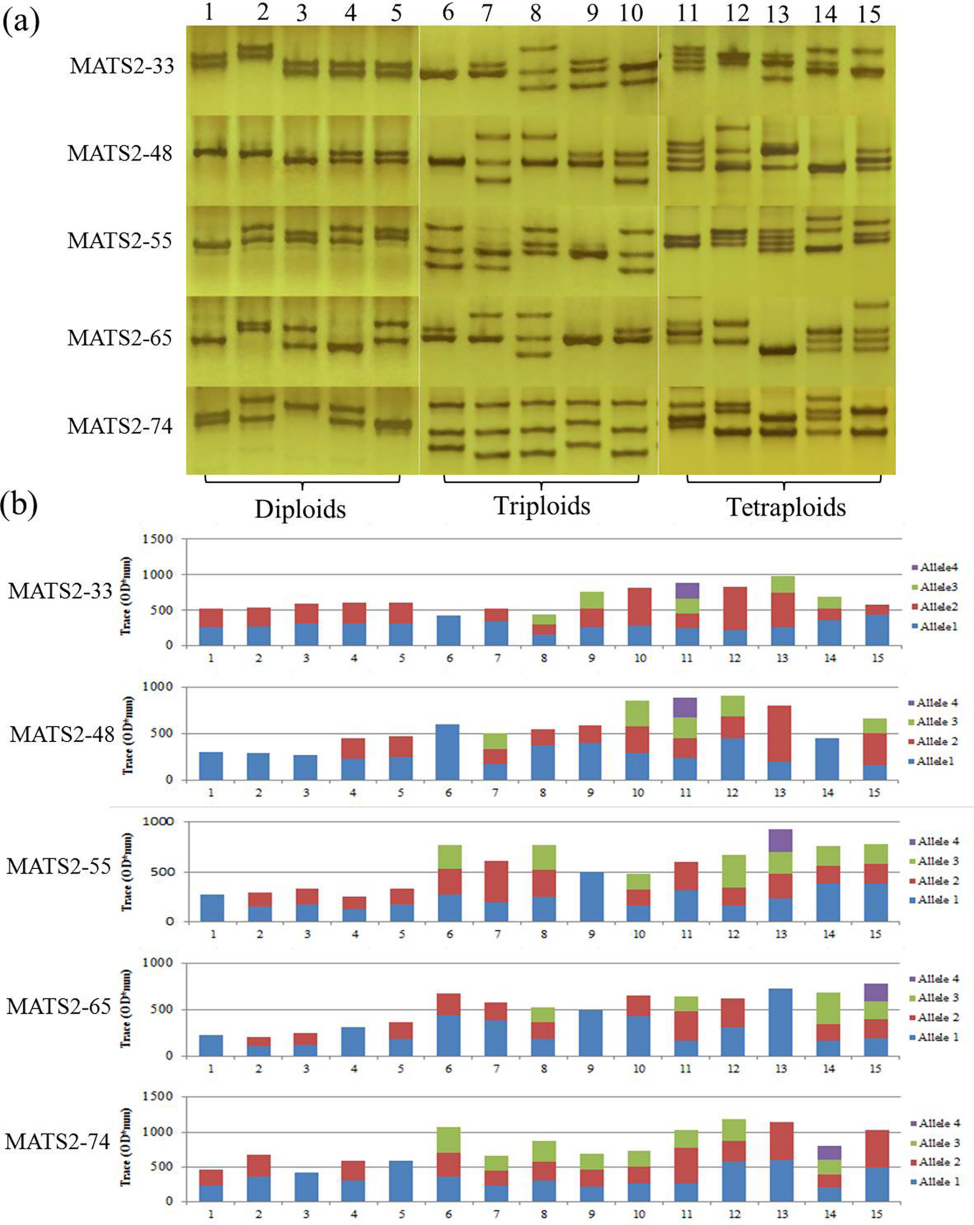
**Examples of microsatellite banding patterns of the reference diploid, triploid and tetraploid *M*. *anguillicaudatus* at loci MATS2-33, MATS2-48, MATS2-55, MATS2-65 and MATS2-74, respectively (a) and the quantitative values corresponding to each allele (from the bottom up) (b)**. Coordinates on *X*-axis (b) refer to the tested samples corresponding to electrophoretic profiles (a), whereas coordinates on the *Y*-axis refers to the allelic quantitative values at each locus provided by the Bio-Rad Quantity One^®^ Bands software.

Sixty-nine reference specimens including 32 diploids, 5 triploids and 32 tetraploids were amplified with the five selected diagnostic markers. Fig 2 showed representative microsatellite banding patterns generated by the five diagnostic markers (a) and their corresponding allelic quantitative values provided by the Bio-Rad Quantity One^®^ software (b). In each locus, ratios between quantitative values for all allele-pairs ranged from 1.00 to 3.12, in accord with the theoretical values of 1.0, 2.0 or 3.0, indicating that these alleles occurred in one, two or three copies, respectively (Table 4).

**Table 4:** Ploidy level estimation of the reference *M. anguillicaudatus* using the five diagnostic ESR-SSR markers^a^.

Locus		1	2	3	4	5	6	7	8	9	10	11	12	13	14	15
**MATS2-33**	Observed-allele No.	2	2	2	2	2	1	2	3	3	2	4	2	3	3	2
	Ratio	1.02/1	1.03/1	1.06/1	1.04/1	1.03/1	—	2.01/1	1.11/1.08/1	1.08/1.09/1	1/1.93	1.09/1.01/ 1/1.01	1/2.93	1.04/1.99/1	2.07/1/1.07	3.01/1
	Calculated-allele No.	2	2	2	2	2	—	3	3	3	3	4	4	4	4	4
**MATS2-48**	Observed-allele No.	1	1	1	2	2	1	3	2	2	3	4	3	2	1	3
	Ratio	—	—	—	1.06/1	1.11/1	—	1.09/1/1.02	2.10/1	2.10/1	1.09/1.08/1	1.10/1.02/ 1.03/1	2.03/1.03/1	1/3.04	—	1.03/1.99/1
	Calculated-allele No.	—	—	—	2	2	—	3	3	3	3	4	4	4	—	4
**MATS2-55**	Observed-allele No.	1	2	2	2	2	3	3	3	1	3	2	3	4	3	3
	Ratio	—	1.06/1	1.09/1	1.00/1	1.06/1	1.11/1.07/1	1/2.11	1/1.10/1	—	1.06/1.03/1	1.09/1	1/1.08/2.02	1.06/1.07/ 1.01/1	2.07/1/1.09	1.94/1/1.03
	Calculated-allele No.	—	2	2	2	2	3	3	3	—	3	2	4	4	4	4
**MATS2-65**	Observed-allele No.	1	2	2	1	2	2	2	3	1	2	3	2	1	3	4
	Ratio	—	1.11/1	1.09/1	—	1.10/1	1.93/1	2.07/1	1.11/1.10/1	—	1.92/1	1.02/2.06/1	1.04/1	—	1/1.07/2.06	1.05/1.11/ 1.05/1
	Calculated-allele No.	—	2	2	—	2	3	3	3	—	3	4	2	—	4	4
**MATS2-74**	Observed-allele No.	2	2	1	2	1	3	3	3	3	3	3	3	2	4	2
	Ratio	1.07/1	1.12/1	—	1.07/1	—	1.09/1/1.06	1.09/1.03/1	1.09/1/1.05	1/1.11/1.09	1.12/1.06/1	1.07/2.07/1	1.91/1/1.03	1.10/1	1.09/1/1.10/1.00	1/1.10
	Calculated-allele No.	2	2	—	2	—	3	3	3	3	3	4	4	2	4	2
**MS-Ploidy**		2n	2n	2n	2n	2n	3n	3n	3n	3n	3n	4n	4n	4n	4n	4n
**FCM-Ploidy**		2n	2n	2n	2n	2n	3n	3n	3n	3n	3n	4n	4n	4n	4n	4n

^a^: Observed-allele No., the number of alleles directly recorded from electrophoretogram; Ratio, observed-allele quantitative values ratio, which was calculated by pairwise combinations between quantitative values for all allele-pairs at a particular locus; Calculated-allele No., the number of alleles obtained by combining the analysis of microsatellite banding patterns and the observed-allele quantitative values ratios; MS-Ploidy, ploidy level estimated by EST-SSRs; FCM-Ploidy, ploidy level determined by flow cytometry. In each locus, we considered the band with the lowest quantitative value representing a single dose allele, and were used as single-copy control for pairwise comparisons.

In a diploid individual, a highly heterozygous SSR should amplify at most two alleles at a particular locus. In triploid and tetraploid individuals, up to three and four alleles could be found, respectively. In fact, only in two cases, the number of alleles cannot be correctly estimated. One possibility is when there is only one unique allele, which cannot provide efficient ratio. The other is genotypes when displaying two distinct alleles but with equal quantitative values, which can happen with both diploids (AB) and tetraploids (AABB). Indeed, we observe some unique alleles (e.g. sample 9 in locus MATS2-65 (Fig 2)) and two distinct alleles (e.g. sample 13 in locus MATS2-74 (Fig 2)). Yet despite all this, we were able to assign putative configurations to the sample using other diagnostic loci that contained a minimum of two alleles.

Specifically, we observed at most two alleles in all diploid individuals (sample 1–5 in Fig 2), while all polyploidy individuals exhibited the maximum allele numbers consistent with their known ploidy levels. For example, the sample 8 had three observed alleles for 4 out of the five EST-SSR markers (Fig 2). The sample 11 had four observed alleles in two EST-SSR markers (Fig 2). If we consult the calculated numbers of alleles, at least 3 out of the five markers exhibited three alleles for the triploid samples (samples 6–10, Fig 2, Table 4), and at least four out of five markers exhibited four alleles for the tetraploid samples (samples 11–15, Fig 2, Table 4).

Thus, based on the maximum calculated-allele number for each individual in the genotyping data matrix (S3 Table), ploidy results estimated for all the reference specimens were as expected: all 32 reference tetraploid specimens producing maximum number of four calculated-alleles at one or more of the five diagnostic loci thus were inferred to be tetraploids, whereas the 32 reference diploids produced two calculated-alleles at most and were identified as diploids. Furthermore, 8 reference triploids from 2 populations were classified as triploids. These results support the utility of the selected EST-SSR loci as a ploidy marker of *M*. *anguillicaudatus*.

### Utility of the diagnostic EST-SSRs for ploidy identification

Ninety-six randomly collected individuals from Liangzi Lake were analyzed to validate the reliability of the five diagnostic EST-SSRs for ploidy identification. In parallel, their ploidy levels were examined using FCM (S4 Table). Based upon the criteria that an individual is considered as triploids or tetraploids if three or four calculated-alleles were observed in at least two EST-SSR loci, the ploidy assignments from these two approaches were 100% concordant in this specific case (Table 5). Nevertheless, some individuals that were considered as 2n could be in fact 4n (for example, sample 13 in S2 Fig), thus utilizing more than two EST-SSR loci will be prudent.

**Table 5:** Results of ploidy identification for randomly collected individuals from Liangzi Lake, China, using two separate methods: EST-SSR loci analysis and flow cytometry^a^.

FCM-Ploidy	n	MS-ploidy		Correct rate	
		2n	4n		
***One locus***					
**2n**	41	37	4	90.24%	95.83%
**4n**	55	0	55	100%	
***At least two loci***					
**2n**	41	41	0	100%	100%
**4n**	55	0	55	100%	

^a^: MS-Ploidy, ploidy level estimated by EST-SSRs; FCM-Ploidy, ploidy level determined by flow cytometry; Correct rate, agreement rate between ploidy determined by flow cytometry and EST-SSRs analyses; *One locus* refers that an individual was considered as triploids or tetraploids if three or four calculated-alleles were observed in one EST-SSR locus, while *At least two loci*, in at least two EST-SSR loci.

## Discussion

In this study, utilizing newly generated transcriptome data and a combination of computational and experimental methods, we demonstrate the utility of EST-SSR markers for ploidy identification. We selected 5 EST-SSR markers to be used for *M*. *anguillicaudatus* individuals. We showed that the ploidy levels of all 69 reference specimens as well as of 96 ploidy-unknown specimens from a mixed ploidy population could be correctly classified, demonstrating the reliability and utility of the diagnostic EST-SSR markers and the associated analytic framework developed in the present study. In addition, as EST-SSRs are generally highly transferable to closely related species [43], the markers developed in this study have the potential to be used to detect ploidy in other loach species.

There are some important considerations that are necessary when developing SSR markers for ploidy identification. Importantly, the efficiency of SSR-assisted ploidy identification depends on the variability of amplified loci [40]. We measured the *PIC* value, which provides an estimate of the variability and discriminatory power of a locus by taking into account, not only the number of alleles that are expressed, but also the relative frequencies of those alleles [44–45]. Gene body derived EST-SSR markers in this study have high *PIC* values (average = 0.78), as well as easy-to-score profile (no or very few stutter bands), enabling us to correctly deduce the allelic configurations in each locus and determine the ploidy of *M*. *anguillicaudatus*.

Population genetic studies in polyploid species using SSR markers are challenging due to the difficulty in defining which allele(s) occurs in more than one copy when the number of displayed microsatellite DNA alleles is less than the possible maximum number for that ploidy level [46–47]. Several studies based on allozymes have inferred polyploid genotypes based on the intensity of bands in the zymograms [48–49]. To estimate the allelic configuration of microsatellite loci in polyploid plants, the Microsatellite Allele Counting-peak ratios method (MAC-PR) has been developed [42], which makes use of the quantitative values for allele amplification peak areas provided by the sizing software [42]. Additionally, direct codominant interpretation of microsatellite loci based on relative PCR product intensities has also been reported [46, 50]. However, these methods depend on the Genetic Analyzer, a highly specialized equipment, which is costly to purchase and maintain, thus not routinely available. In the current study, allelic dosage was resolved by estimating the number of alleles in a band according to the relative intensity profile of the bands. The copy number of an allele can be estimated based on the quantitative allele amplification band intensity provided by the Quantity One software, which is freely available on the Bio-Rad web site (http://www.bio-rad.com).

Another potential limitation of SSRs in ploidy identification is possible null alleles, which fail to amplify due to primer site variation [20, 25, 51]. Null alleles, when segregating with another allele, can lead to underestimation of the number of alleles. The five diagnostic EST-SSR markers developed in this study do not show any evidence of null alleles in the 165 samples analyzed (S3 and S4 Tables). This could be due to (i) this set of microsatellite specifically developed from the transcriptome of this species itself because, usually, the frequency of null alleles rapidly increases when SSR primers are transferred to other species [47]; (ii) the primers flanking of EST-SSRs are derived from relatively conserved sequences, therefore, it is likely that if present they should be in very low frequencies [47, 51–52]. Additionally, it has been proposed that when an appropriate number of high variable SSRs is employed, null alleles does not present a significant problem in ploidy identification since the large amount of variability at microsatellite loci compensates the potential biases introduced by any one locus [53–54]. Although we cannot totally discard the presence of null alleles in future samples, the current data suggest that their frequencies are likely to be extremely low, and their effects on polyploidy identification would be minimal.

In summary, we took advantage of next-generation transcriptome analyses to develop a large number of putative EST-SSR markers for *M*. *anguillicaudatus*. Among these, a set of five ploidy diagnostic EST-SSR markers and the associated analytic framework offer a minimally-invasive, reliable and efficient molecular method for identifying the ploidy levels of the loach. This method can be applied to juveniles and scanty fish samples for which karyotyping and flow cytometry are difficult, thus would be a valuable addition to the tools available for ploidy identification of *M*. *anguillicaudatus*.

## Supporting information

S1 Fig Length distribution of all the unigenes generated by *de novo* assembly. (TIF)

S2 Fig Gel images of the 69 reference specimens and 96 ploidy-unknown specimens. (DOCX)

S1 Table Characteristics of 45 EST-SSR markers validated in a survey of 32 diploid individuals from transcriptome sequences of *Misgurnus anguillicaudatus*. (XLSX)

S2 Table Characteristics of the EST-SSR loci and their corresponding primer pairs developed from *Misgurnus anguillicaudatus* transcriptome sequences. (XLSX)

S3 Table Ploidy level estimation of 69 reference *Misgurnus anguillicaudatus* using the five diagnostic ESR-SSR markers. (XLS)

S4 Table Ploidy level estimation of 96 randomly selected *Misgurnus anguillicaudatus* from Liangzi Lake, China, using two separate methods: EST-SSR markers and flow cytometry. (XLS)
