# Supplementary figures and images for: Development of novel EST-SSR markers for ploidy identification based on de novo transcriptome assembly for Misgurnus anguillicaudatus
Source: PLoS One. 2018 Apr 12;13(4):e0195829. doi: 10.1371/journal.pone.0195829 (PMC5896994; doi:10.1371/journal.pone.0195829)

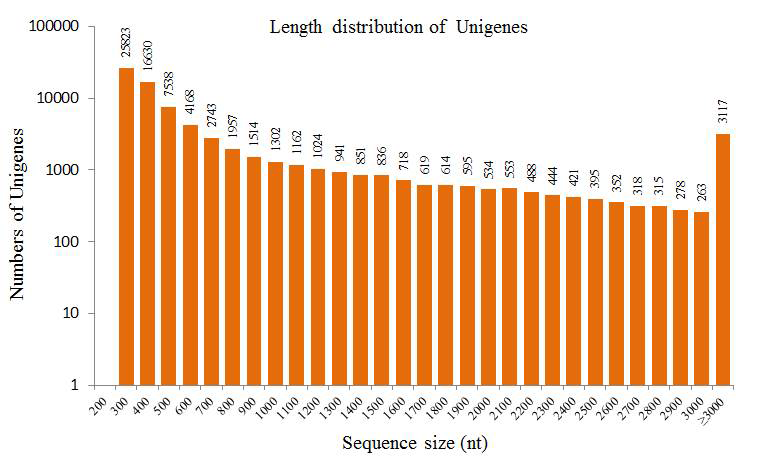

Supplement: S1 Fig — (TIF) [file pone.0195829.s001.tif]
