# Supplementary material for: Development of novel EST-SSR markers for ploidy identification based on de novo transcriptome assembly for Misgurnus anguillicaudatus
Source: PLoS One. 2018 Apr 12;13(4):e0195829. doi: 10.1371/journal.pone.0195829 (PMC5896994; doi:10.1371/journal.pone.0195829)

**Gel images of the 69 reference specimens and 96 ploidy-unknown specimens**

(Specimens in the red boxes were used as examples and shown in manuscript)

4N (32 reference specimens)


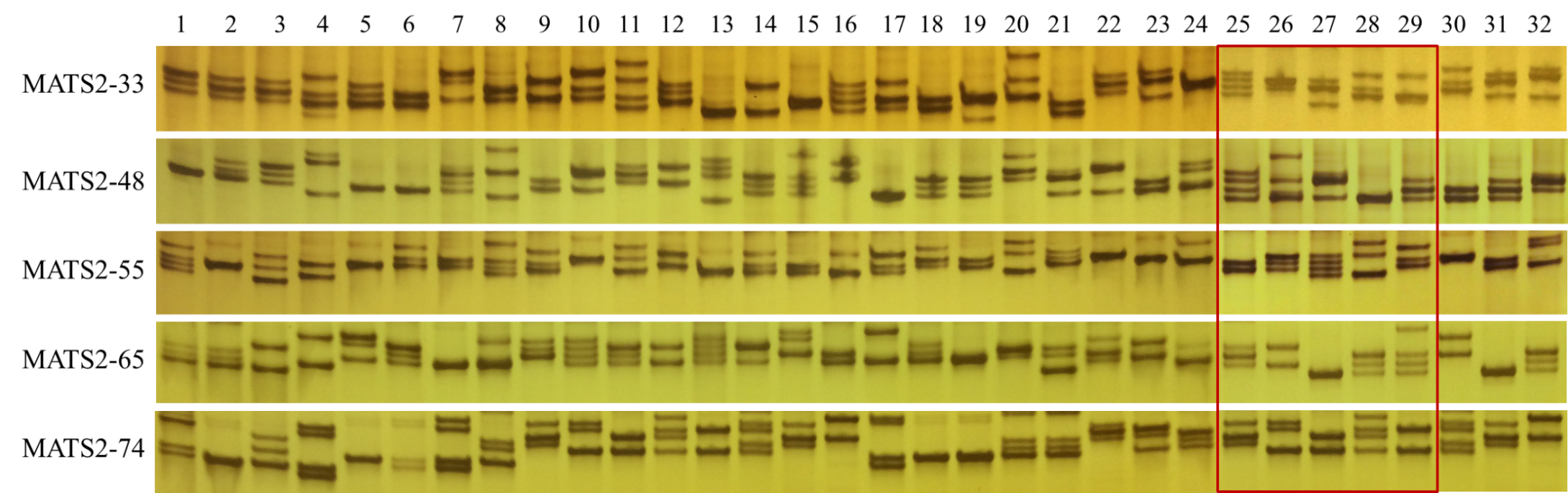


3N (5 reference specimens)


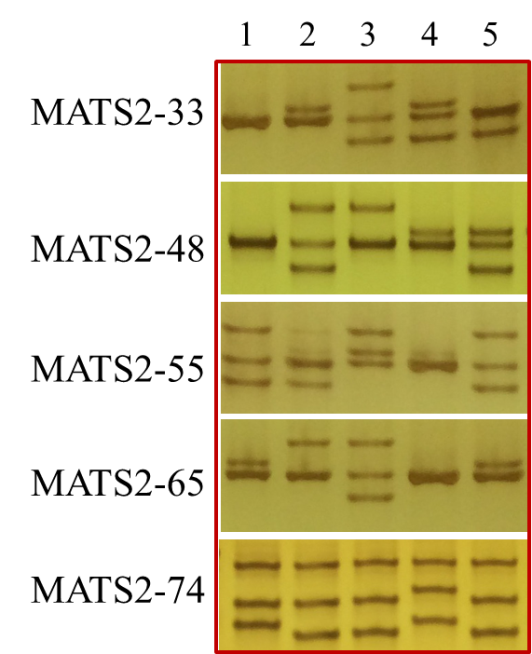


2N (32 reference specimens)


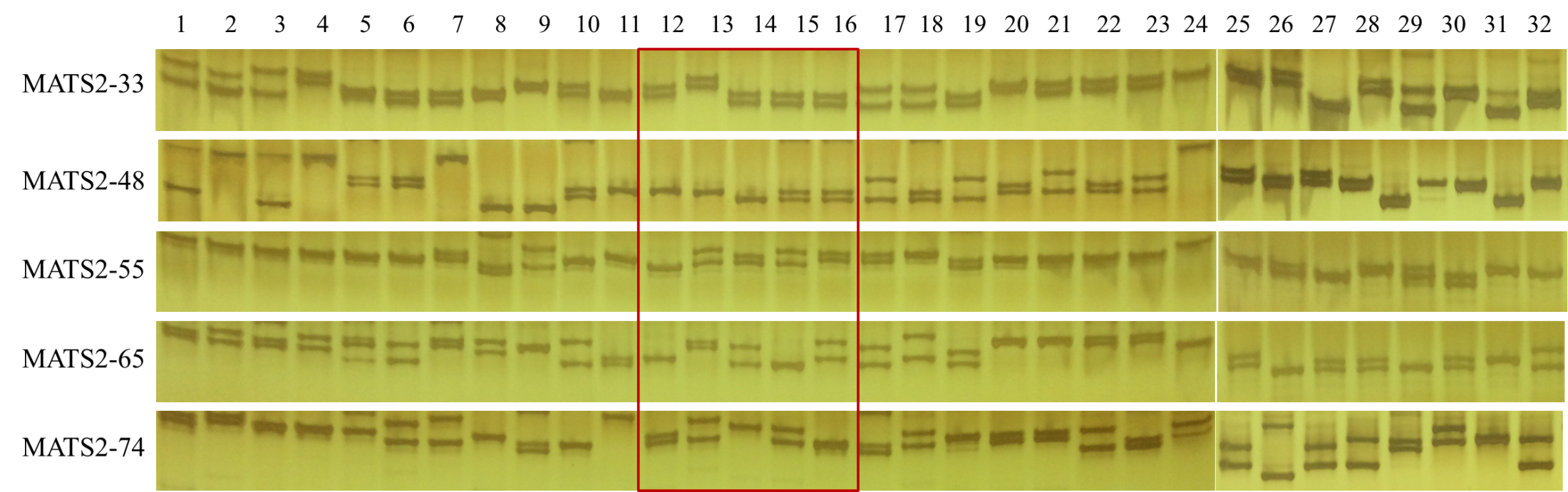


96 ploidy-unknown specimens

Specimens 1-24


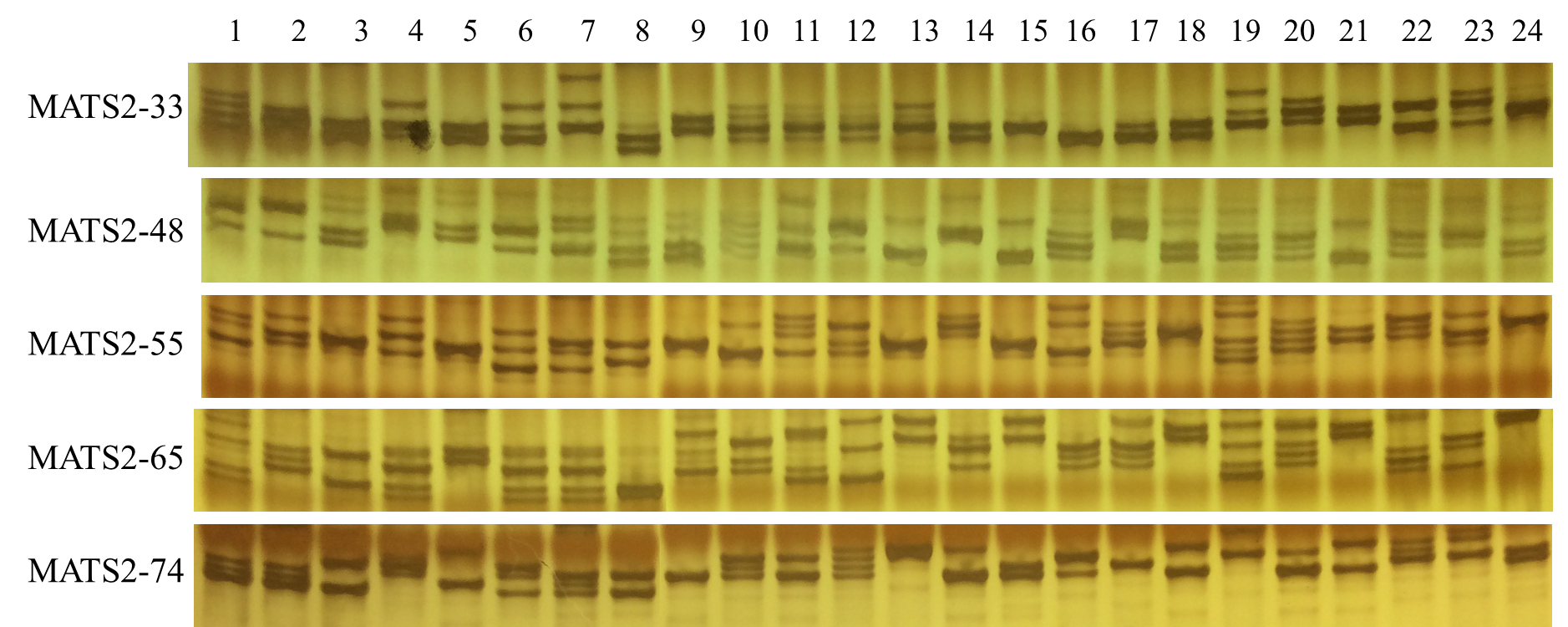


Specimens 25-48


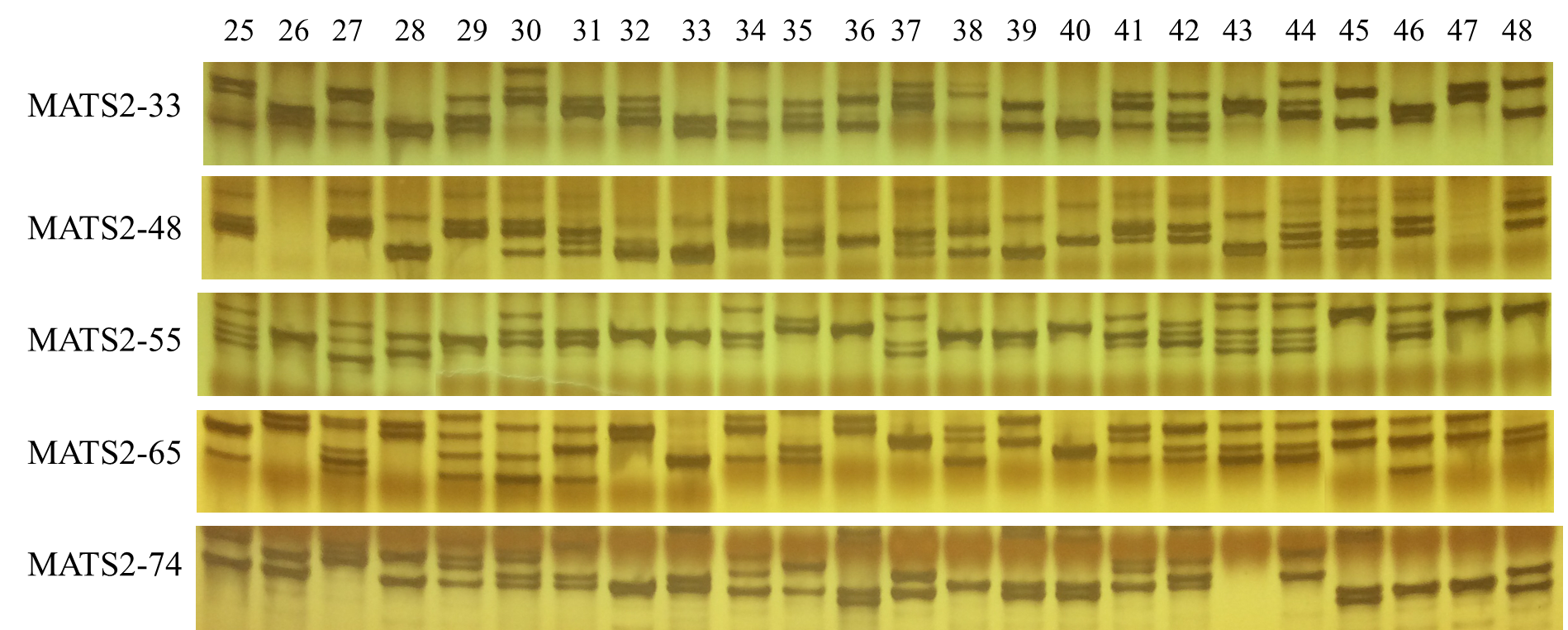


Specimens 49-72


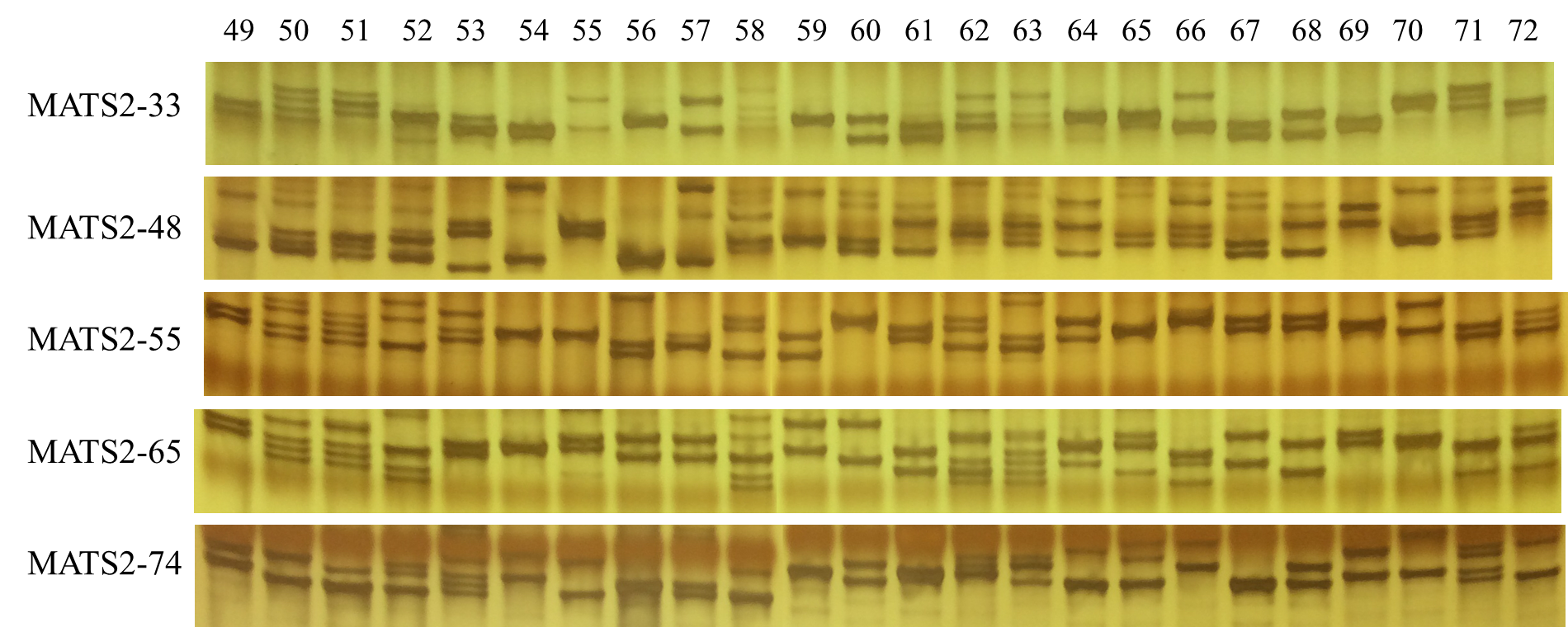


Specimens 73-96


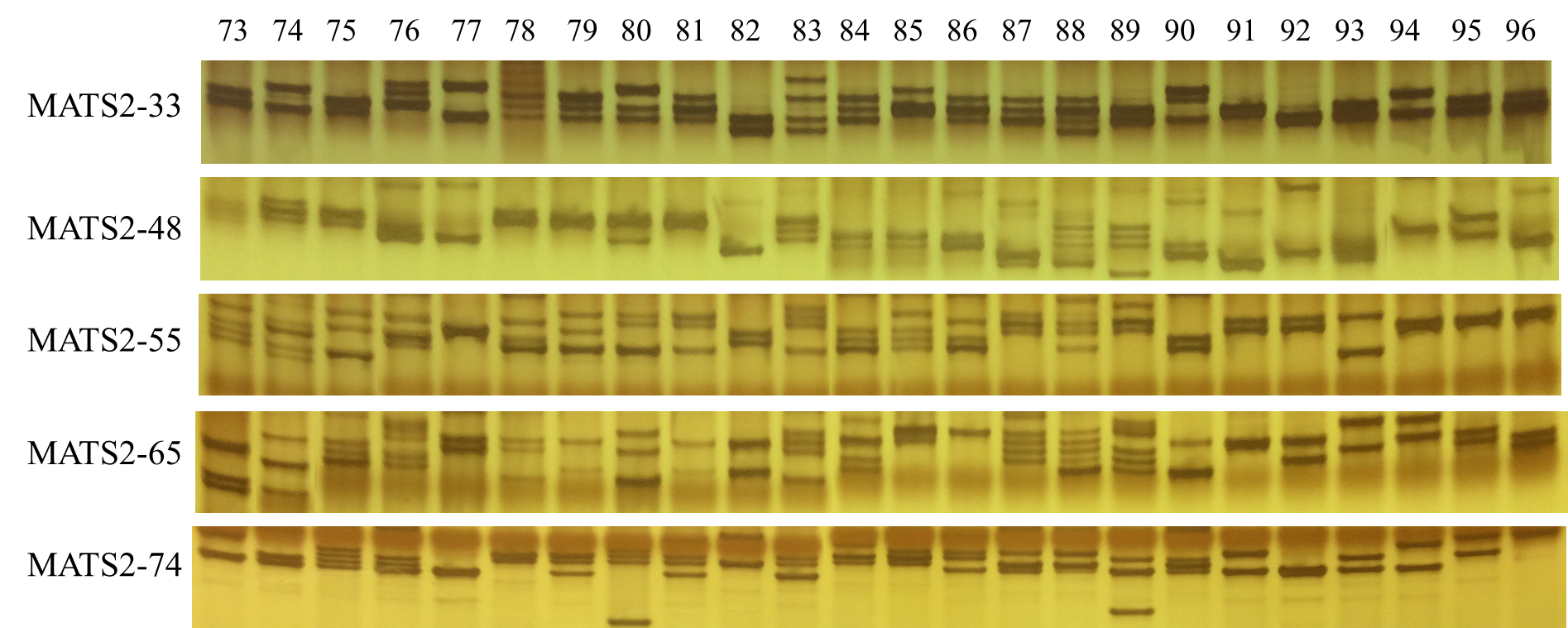

Supplement: S2 Fig — (DOCX) [file pone.0195829.s002.docx]
